# The efficacy of transdiagnostic cognitive behavioral therapy on migraine headache: a pilot, feasibility study

**DOI:** 10.1186/s12883-022-02729-8

**Published:** 2022-06-22

**Authors:** Forouzandeh Soleimanian-Boroujeni, Negin Badihian, Shervin Badihian, Vahid Shaygannejad, Yousef Gorji

**Affiliations:** 1 Department of Psychology, Khomainishahr Branch, Islamic Azad University, Khomainishahr, Isfahan, Iran; 2 Isfahan Neurosciences Research Center, Alzahra Research Institute, Isfahan University of Medical Sciences, Isfahan, Iran; 3 Department of Neurology, Johns Hopkins University School of Medicine, Baltimore, MD USA

**Keywords:** Cognitive Behavioral Therapy, Migraine Disorders, Headache, Clinical trial, Transdiagnostic therapy

## Abstract

**Introduction:**

Psychological interventions are shown to be effective in migraine, but not utilized routinely yet. We aimed to evaluate the efficacy of transdiagnostic cognitive behavioral therapy (TCBT) on people with migraine (PwM).

**Method:**

This study was conducted on 40 PwM aged 20–50 years. We randomly assigned participants to two groups of intervention, receiving 10 sessions of TCBT, and control, attending one session on relaxation and stress-management techniques. Days with headache, headache severity, migraine-related disability and effects on daily life, number of pain-relivers taken for headache, depression, and anxiety were assessed pre-intervention, post-intervention (three-month follow-up), and one-month after TCBT termination (four-month follow-up).

**Results:**

Thirty-five participants suffering moderate to severe migraine completed the study (16 and 19 in TCBT and control groups, respectively). TCBT improved all measured items between study time-points (*p* < 0.05) in the intervention group, while such an improvement was not observed in the control group. Between group comparisons revealed superiority of TCBT group compared to the control group in most measured items at three- and four-month follow-ups (*p* < 0.05).

**Conclusion:**

Ten sessions of TCBT improved migraine severity, associated disability, anxiety, and depression in PwM, with persistent effects after one month of therapy termination. However, the generalizability of these findings is limited due to the placebo effect in the intervention arm, given the more time each participant has spent with the therapist. TCBT could be an affordable, practical, and feasible intervention to be utilized for PwM and larger studies with equal number of sham therapy sessions are needed to further explore this.

**Trial registration number:** The study protocol was registered in clinicaltrial.gov (NCT03701477) prior to enrollment.

## Introduction

Migraine is among the most prevalent primary headache disorders and one of the most important causes of disability worldwide [1]. It presents with recurrent attacks of moderate to severe headache lasting 4–72 h. The global prevalence of migraine has been reported to be 11.6% in 2017 [2]. Migraine can affect both private and social aspects of life and lead to decreased quality of life. It mostly affects young to middle age population, hence, brings significant direct and indirect costs and is an important economic burden to the society. In 2016, the annual costs of migraine per person in the United States, due to using healthcare resources and productivity losses, were reported to be around $8,243 and $2,649 for chronic and episodic migraines, respectively [3]. The prevalence of psychological conditions, such as depression, anxiety, and mood disorders, is shown to be higher in people with migraine (PwM). These psychological conditions exacerbate the disability caused by migraine and would also increase the risk of progression to chronic form of the disease [4, 5]. Current therapeutic approaches for migraine treatment mostly include using medications for prophylaxis and acute attacks. Regardless of the side effects associated with these medications, yet many PwM suffer from recurrent attacks even with regular usage of prophylactic medications [6]. Moreover, adherence to prophylactic migraine medications is shown to be low, mostly because of the side effects [7, 8]. This could further deteriorate the condition and associated disability.

Some investigators have suggested migraine as a biopsychosocial condition that would respond well to psychological interventions at various stages [9]. A systematic review conducted in 2016 showed the efficacy of psychological interventions in migraine [10]. These psychological interventions include cognitive-behavioral therapy (CBT), relaxation trainings, stress management, massage therapy, etc. [9, 11]. CBT is reported to be efficacious in decreasing the frequency and severity of headaches in PwM [10, 11]. However, delivering CBT faces multiple challenges: high expenses, presence of multiple comorbidities that can confuse both the therapist and the patient, inadequate resources, and limited number of trained psychologists in these techniques. Therefore, diagnostic-based psychological interventions such as CBT are less feasible and practical in the real world, despite being efficacious [10–12].

To address the feasibility and practicability issues, Transdiagnostic CBT (TCBT) could be considered. TCBT is a technique that focuses on therapeutic modules that target the shared underlying mechanisms across diseases (see Table 1 for an exemplary scheme of the technique) [13, 14]. When delivering TCBT, the therapist does not need to focus on a specific psychological diagnosis and can implement a predetermined protocol for various psychological complaints. This means easier training of therapists and more resources for care delivery. A study has shown the effectiveness of TCBT on improving headache and psychological disturbances (e.g., anxiety and depression) among adolescents. [15]. No studies have evaluated the efficacy of TCBT on adult population with migraine, hitherto. Considering the high prevalence of comorbid anxiety and depressive symptoms among PwM and the efficacy of TCBT in these psychological conditions and headache in adolescents, we aimed to investigate the efficacy of TCBT on headache and the accompanying symptoms in a group of adult PwM. We hypothesized that TCBT can effectively improve migraine and decrease the associated disability and impact on PwM’s lives.

**Table 1:** Description of activities in each therapy session

**First session**
**Therapeutic agreement and psychoeducation**
1. Forming a therapeutic agreement between patients and the therapist regarding number of therapy sessions and their durations, the process of treatment, and confidentiality issues
2. Educating patients about the nature and causes of headaches and its comorbid psychological disorders and discussing definition of anxiety
3. Explaining the importance of completing homework at home, and providing instructions on homework,
4. Giving homework to patients on self-monitoring (recording dates of experiencing headaches, duration of the pain, and its severity based on a scale from 0 to 10; rating the average daily anxiety level on a scale of 0 to 100)
5. Asking patients to provide a list of situations that are associated with distressing anxiety for them
6. Briefly explaining the content of the next session
**Second session**
**Educating relaxation training and hierarchy of behavioral avoidance**
1. Educating relaxation techniques and preparation of hierarchy of behavioral avoidance for the better management of headache and anxiety responses
2. Evaluating patients’ homework on self-monitoring and analyzing headache triggers
3. Explaining the components of anxiety to patients
4. Giving homework to patients on relaxation exercises
5. Giving homework to patients on self-monitoring
6. Briefly explaining the content of the next session
**Third session**
**Automatic negative thoughts and guided imagery**
1. Evaluating patients’ problems in practicing relaxation techniques and preparing a hierarchy of behavioral problems as an important part of the treatment
2. Discussing the importance of thoughts and educating how to identify automatic negative thoughts
3. Giving homework to patients on recognizing and recording automatic negative thoughts
4. Evaluating headache and anxiety daily diary recorded by the patient
5. Delivering a CD containing audio on guided imaginary to patients to practice with at home
6. Briefly explaining the content of the next session
**Fourth session**
**Cognitive restructuring**
1. Ensuring that the patients have performed their self-monitoring homework and relaxation exercises
2. Assessing automatic thoughts identified by patients during the past week
3. Educating patients on how to challenge automatic thoughts
4. Educating patients on cognitive errors and helping them to identify such errors
5. Addressing patients’ ambiguities
6. Briefly explaining the content of the next session
**Fifth session** **Problem solving skills and distraction technique**
1. Evaluating patients’ improvements in performing homework
2. Educating problem solving skill and using role-playing techniques
3. Educating distraction technique to reduce overthinking
4. Giving homework on problem solving and distraction skills
5. Briefly explaining the content of the next session
**Sixth session**
**Exposure and cognitive restructuring**
1. Evaluating problem solving homework, assessing learned skills and the way patients apply them in their daily lives
2. Educating self-directed exposure to patients and giving them homework on this skill
3. Educating advanced cognitive reconstruction steps and encouraging patients to apply them in real life situations
4. Extensive focusing on daily general stress level instead of targeting a special problem
5. Summarizing the session and addressing patients’ ambiguities
6. Briefly explaining the content of the next session
**Seventh, Eighth, and Ninth session**
**Advanced cognitive restructuring, stress management, assertiveness skills training, preparation for therapy termination**
1. Evaluating self-monitoring homework, exposure, and cognitive reconstruction of patients to ensure their proper learning and applying them by patients in their lives
2. guiding patients in cognitive reconstruction processes
3. Educating stress management, assertiveness skills, and the ability to say “No”
4. Advising patients to continue cognitive reconstruction homework, stress management techniques, and assertiveness skills in appropriate situations
5. preparing patients for the last therapy session
**Tenth session**
**Relapse prevention and therapy termination**
1. Evaluating patients’ performances in stress management and assertiveness skills
2. Encouraging patients to continue doing homework and rewarding themselves
3. Educating patients to administer the learned therapeutic techniques in case of disease recurrence
4.Summarizing previous sessions and getting feedback from patients on different therapy sessions and effectiveness of the learned techniques
5. Discussing with patients about the new perspectives they need to learn about themselves and their problems
6. Congratulating patients on completing therapy sessions and acknowledging their participation in the program

## Materials and method

### Study participants

This is a pilot randomized clinical trial conducted during October 2018 to April 2019 at a single outpatient private counselling clinic in Isfahan, Iran. The inclusion criteria were: 1) The diagnosis of migraine headache by the primary neurologist based on the criteria defined by International Classification of Headache Disorders III Beta (ICHD-III Beta) [16]; 2) Diagnosed with migraine at least 6 months prior to the enrollment; 3) High school graduate or higher level of education (in order to incorporate trainings during therapy); 4) Aged 20–50 years. Patients were excluded from the study in case of medication overuse diagnosed based on the criteria defined by ICHD-III Beta (taking non-steroidal anti-inflammatory drugs or other pain relievers at least 15 days in each month; taking triptans or similar drugs at least 10 days each month for 3 months or more) [16]; using new prophylactic migraine medication during the study; suffering from other types of headache disorders in addition to migraine (e.g. cluster headaches, headaches due to structural abnormalities, etc.); undergoing any other psychological therapies during the study (not considering psychiatric medications that are used for migraine); and altered cognitive or mental health status (like dementia). Patients were initially recruited in the study neurologist’s office, and then referred to the study team for further evaluations. The study procedure was explained to the participants and written informed consents were obtained from all participants. With a power of 80%, α of 0.05, and effect size of 0.9, we estimated 16 participants are needed in each group to show a significant effect of the intervention. The effect size was estimated using HIT scores in the study by Sharma et al. [15]. However, we decided to recruit 20 participants in each group given the possibility of dropouts or withdrawals. The study was approved by the ethics committee of Islamic Azad University of Khomeinishahr (ID:18,820,701,962,055) and was registered in clinicaltrials.gov prior to enrollment (ID: NCT03701477, posted on 10/10/2018).

### Study design

After obtaining informed consents and prior to randomization, we asked all participants to fill out the study questionnaires, explained below under “Outcome measures”. We used random allocation software in four blocks to generate random numbers and divided participants into study groups. The intervention group underwent 10 sessions of TCBT (Table 1). The control group were scheduled for a single 3-h meeting in which basic techniques of relaxation and stress management were discussed. All patients were also encouraged to adhere to their current migraine treatment and not to start new medications or therapies or discontinue current regimen, unless advised by their neurologist.

One week after the last therapy session of the intervention group, we asked the participants to fill out the same questionnaires for the second time as post-intervention measurement (3-month follow-up). In order to assess persistence of the effects of therapy sessions, we asked patients to come back one month after the last therapy session and complete the questionnaires for the third time (4-month follow-up). The control group completed the questionnaires on similar time-points. The primary investigators and outcome assessor were masked to the randomization status.

### Intervention

The therapy protocol was derived from the suggested protocol by Sharma et al. on TCBT intervention for headache in adolescents [17]. We conducted 10 sessions of group based TCBT, each session lasting for 2 h, by a trained, experienced psychologist. The sessions were held regularly at a definite time and place for continuous weeks. There was an exception for the last session which was held with a 2-week gap to give patients some extra time to incorporate what they have learned into their problems. We gave specific homework papers to the participants after each session. The homework included pre-specified tasks to practice what has been discussed during that session, practices to incorporate the new skills they have learned, and using these skills to deal with their problems. Patients were asked to do their homework and return it the next session. At the beginning of each therapy session, participants’ homework papers were reviewed by the therapist and new tasks and skills were explained to the participants. At the end of each session, patients were asked to summarize the new skills they have learned and provide feedback on the new skills. Detailed description of topics and skills discussed in each session is presented in Table 1.

### Outcome measures

Sociodemographic characteristics of participants including age, sex, education, number of days with headache within the past 30 days, headache severity, and history of medications used for migraine headache (both prophylactic medications and pain relievers) were recorded at baseline. To collect data on days with headache and the number of used medications, we asked participants to write those down in a headache diary.

We assessed participants at three time-points: baseline (prior to intervention), at the end of the last therapy session (3-month follow-up), and after 1 month of therapy termination (4-month follow-up). In each time-point, patients were evaluated regarding the level of pain, migraine effects on the individual’s function in different aspects of life, migraine disability, depression, and anxiety.

For the assessment of pain severity, we used Wong-Baker Faces Pain Rating Scale (WBFPRS), also known as visual analog scale (VAS) [18]. It is a well-known scale consisted of six faces that represent the effect of pain on emotion. This instrument could be scored from 0 (no pain at all) to 10 (severe pain). For using this instrument, we asked patients to show the face that could better describe them during pain. Headache Impact Test-6 (HIT‐6) was used to assess migraine effects on the individual’s function [19]. HIT-6 includes 6 questions evaluating different aspects of patient’s life that may be affected by migraine. Each question is scored through Likert system and can be scored from 6 (never) to 13 (always). The total score of the questionnaire would be calculated by summing up scores from each single question. Higher scores indicate more severe migraine and scores over 50 show migraine has notably disturbed patient’s daily activity [20]. The study was powered to HIT-6 as the primary outcome measure.

Migraine Disability Assessment Scale (MIDAS) was used to determine number of days patients have lost during the past 3 months due to the disability caused by migraine [21]. It contains 7 questions and by summing up the number of lost days written by the patient for 5 of the question, the disability would be assessed. Using this questionnaire, patients are classified to have no/very little disability (0–5), mild disability (6–10), moderate disability (11–20), and sever disability (≥ 21) [22]. The other 2 items are known as MIDAS-A and MIDAS-B and assess headache frequency (number of days spent with migraine headache during the past 3 months), and the average pain intensity (on a scale of 1–10), respectively. These 2 items are not accounted in measuring the total MIDAS score.

Hospital Anxiety and Depression Scale (HADS) was used to evaluate the level of anxiety and depression [23]. This scale contains 7 questions on anxiety and 7 on depression. Each question can be scored from 0 to 3 based on a Likert system. The total score for anxiety and depression is calculated by adding up the scores of each single question. This scale classifies patients to 3 different categories of normal, borderline, and abnormal anxiety and/or depression [24]. We used the validated Persian version of all these instruments, as cited above.

### Statistical analysis

We described the results using mean values ± standard deviation (SD) or frequencies (%). Independent t-test or the non-parametric equal were used to compare baseline interval characteristics between two groups. We used Chi-square test to compare categorical data between two groups. Primary and secondary outcomes were assessed in each group using repeated measure analysis of variance (ANOVA). We used Greenhouse–Geisser correction in case sphericity was violated. The sphericity was evaluated using Mauchly’s test of sphericity. In case of any statistically significant findings, we used post-hoc Bonferroni test to distinguish where the difference is. Prior to repeated measure ANOVA, Pearson correlation was used to identify possible covariates to be included in the analysis. We also compared the outcome measures as categorical data between two groups and in each group. The categories are previously explained under “outcomes measures”. Comparing these categorical outcome measures between groups was done using Chi-square test. To compare the categories in each single group between different time-points, we used Friedman test. To compare the outcome measures between two groups in each time-point separately, we used one-way analysis of covariance (ANCOVA), adjusting for the amounts reported in the baseline visit. For example, scores at three- and four-month follow-up were compared between two groups adjusting for the baseline scores. We used Statistical Package for Social Sciences software (SPSS, Armonk, NY; version 25) for statistical analysis and a p-value of less than 0.05 was considered as statistically significant.

## Results

### Recruitment and baseline data

**Fig. 1:**
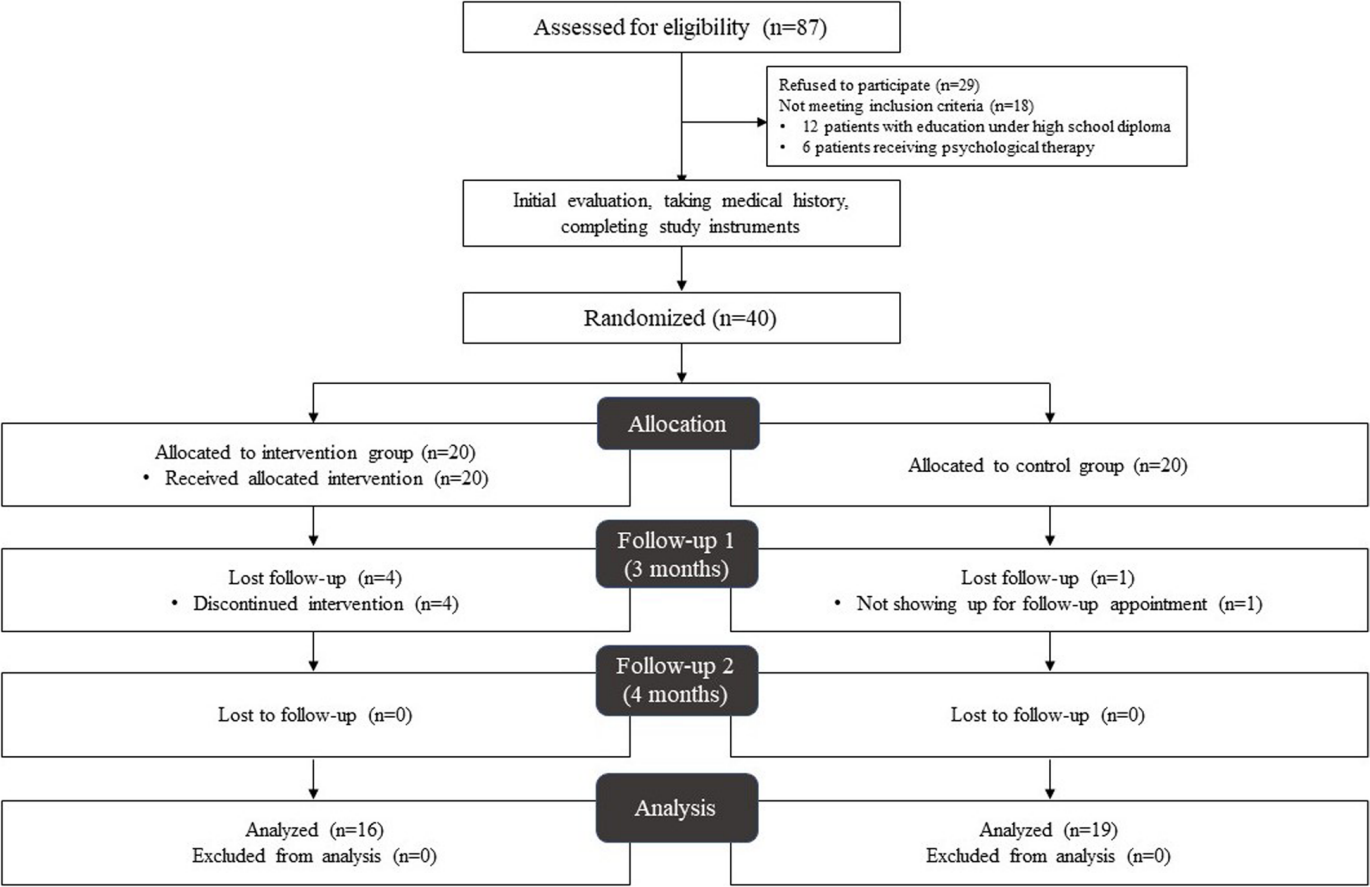
Study diagram

We approached 87 patients, 18 patients were not eligible for the study (12 patients had educations under high school diploma, 6 were receiving psychological therapy) and 29 patients did not agree to participate in the study. Hence, 40 patients were enrolled and randomly assigned to intervention (TCBT) or control groups. We lost 4 patients in the intervention group before the 3-month follow-up, due to time limitations and not being able to maintain therapy. One patient in the control group did not show up for 3-month and 4-month follow-ups. Figure 1 shows the study diagram. Table 2 presents baseline characteristics of two study groups. The mean age of patients in the intervention and control group were 37.1 ± 9.7 and 37.3 ± 9.2, respectively (*p* = 0.937). Female patients comprised 93.8% (15 cases) of the intervention group and 89.5% (17 cases) of the control group (*p* = 0.653). The mean migraine duration was 16.2 ± 10.9 years in the intervention group and 9.3 ± 8.1 years in the control group (*p* = 0.063).

**Table 2:** Baseline characteristics in two study groups

**Category**		**Intervention group** **(*N* = 16)**	**Control group** **(*N* = 19)**
Gender	Female	15 (93.8%)	17 (89.5%)
	Male	1 (6.2%)	2 (10.5%)
Age (years)- Mean (standard deviation)		37.1 (9.7)	37.3 (9.2)
Migraine duration (years)- Mean (standard deviation)		16.2 (10.9)	9.3 (8.1)
Education	High school diploma	7 (43.8%)	8 (42.1%)
	Bachelor’s degree	5 (31.3%)	7 (36.8%)
	Master’s degree	3 (18.8%)	3 (15.8%)
	PhD or equivalent	1 (6.3%)	1 (5.3%)
Job	Unemployed/ Housewife	10 (62.5%)	8 (44.4%)
	Student	1 (6.3%)	2 (11.1%)
	Teacher	1 (6.3%)	2 (11.1%)
	University professor	1 (6.3%)	1 (5.6%)
	Accountant	1 (6.3%)	0
	Manager	1 (6.3%)	0
	Self-employed	0	2 (11.1%)
	Other	1 (6.3%)	3 (16.7%)
	Prefer not to say	0	1 (5.6%)
Migraine medications	Sodium valproate, 200 mg or 400 mg daily	13 (81.3%)	18 (94.7%)
	Maprotiline, 20 mg daily	7 (43.8%)	5 (26.3%)
	Verapamil, 20 mg daily	4 (25%)	7 (36.8%)
	Propranolol, 20 or 40 mg daily	4 (25%)	10 (52.6%)
	Sertraline, 50 mg daily	2 (12.5%)	4 (21.1%)
	Nortriptyline, 20 mg daily	1 (6.3%)	0
	Topiramate, 200 mg daily	1 (6.3%)	0
	Doxepin, 10 mg daily	1 (6.3%)	0
	Metoprolol, 40 mg daily	1 (6.3%)	1 (5.3%)
	Amitriptyline, 20 mg	0	1 (5.3%)
	Citalopram, 20 mg	0	1 (5.3%)
Other diseases	Gastroesophageal reflux disease	1 (6.3%)	0
	Hyperlipidemia	1 (6.3%)	1 (5.3%)
	Hypothyroidism	0	1 (5.3%)
	Inflammatory bowel disease	1 (6.3%)	0
	Mild asthma	0	1 (5.3%)
	Multiple sclerosis	1 (6.3%)	0
Mostly used pain relievers	Acetaminophen	0	1 (5.3%)
	Diclofenac (supp)	1 (6.3%)	2 (10.5%)
	Ibuprofen (tab)	6 (37.5%)	9 (47.4%)
	Ibuprofen (cap)	0	6 (31.6%)
	Mixed pain relievers (acetaminophen, ibuprofen, caffeine)	9 (56.3%)	0

### Between group comparisons

There were no statistically significant differences in frequency of categories of MIDAS, HIT, HADS-anxiety, and HADS-depression between two study groups at baseline (*p* = 0.797, *p* = 0.352, *p* = 0.959, and *p* = 0.511, respectively).

**Fig. 2:**
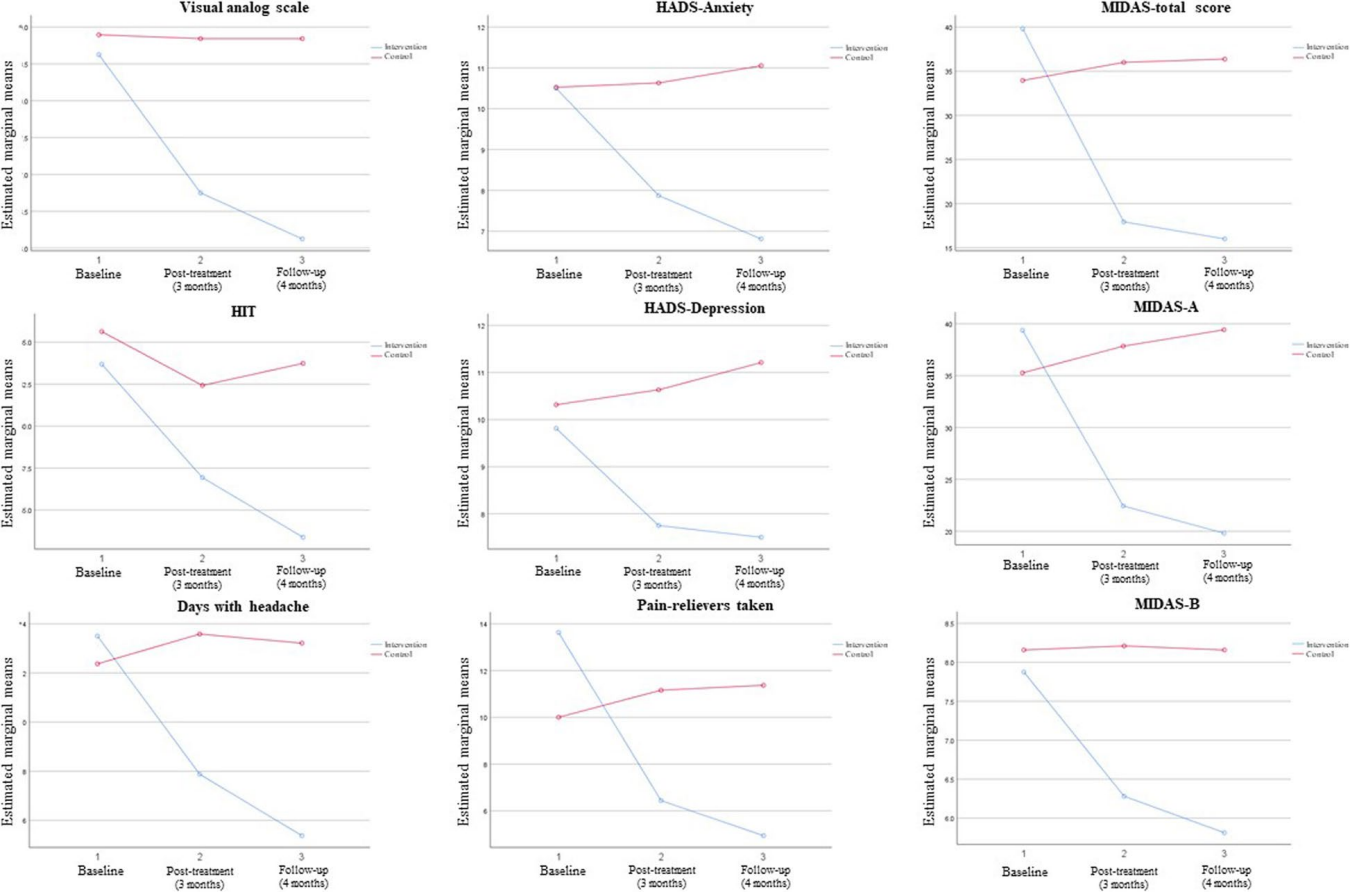
Comparison of mean scores from study instruments and outcome measures in three study time-points. HIT: Headache Impact Test; Days with headache: Number of days the patients has experienced headache within the past 30 days; Pain relievers taken: Number of pain-relievers taken during the past 30 days; HADS: Hospital Anxiety and Depression Scale; MIDAS: Migraine Disability Assessment Scale. Estimated marginal means evaluated using repeated measure analysis of variance

We used repeated measure ANOVA test to assess any interaction between assigned study group and observed outcomes. We found a statistically significant interaction between type of intervention and the scores/values from VAS, MIDAS (total, A, and B), HIT, HADS (both anxiety and depression), days with headache within the past 30 days, and number of pain-relivers taken during the past 30 days (*p* < 0.05). Figure 2 illustrates the comparison graphs on these outcome measures.

Moreover, we compared the outcome measures, both in numbers and in categories, between two groups using ANCOVA and Chi-square, as appropriate. These results are presented in Table 3. As demonstrated in the table, we observed improvement in days with headache, number of pain-relievers taken, VAS, MIDAS (total, A, and B) scores, and HADS (both anxiety and depression) scores immediately after intervention. However, we found no statistically significant difference in MIDAS categories, HIT scores and categories, and HADS anxiety categories between two groups at this time-point. On four-month follow-up, all these outcome measures showed a statistically significant difference between two groups, when adjusting for baseline scores.

**Table 3:** Mean scores from study instruments and outcome measures in three study time-points

**Category**		**Baseline**			**3-month follow-up**			**4-month follow-up**		
		**Intervention** **(N-16)** Mean (SD)/ N (%)	**Control** **(*N* = 19)** Mean (SD)/ N (%)	***P*-value**^**1**^	**Intervention** **(N-16)** Mean (SD)/ N (%)	**Control** **(*N* = 19)** Mean (SD)/ N (%)	***P*-value**^**2**^	**Intervention** **(N-16)** Mean (SD)/ N (%)	**Control** **(*N* = 19)** Mean (SD)/ N (%)	***P*-value**^**2**^
Days with headache (days)		13.5 (6.1)	12.4 (7.4)	0.341	7.9 (4.3)	13.6 (8.1)	0.001^*^	5.4 (2.8)	13.2 (8)	< 0.001^*^
Pain-relievers (number)		13.6 (6.7)	10 (5.2)	0.080	6.4 (4.6)	11.2 (6.9)	< 0.001^*^	4.9 (3.1)	11.4 (7.1)	< 0.001^*^
Headache severity-VAS		8.6 (1.4)	8.9 (1.2)	0.603	6.8 (1.6)	8.8 (1.3)	< 0.001^*^	6.1 (1.4)	8.8 (1.3)	< 0.001^*^
MIDAS-total score		39.8 (19.4)	34 (23.7)	0.174	17.9 (15.9)	36 (24.8)	< 0.001^*^	16 (16.5)	36.4 (25.1)	< 0.001^*^
MIDAS-A		39.4 (17.1)	35.3 (19.6)	0.517	22.4 (14.2)	37.8 (22.2)	< 0.001^*^	19.8 (13.6)	39.4 (21.1)	< 0.001^*^
MIDAS-B		7.9 (1.6)	8.2 (1.2)	0.494	6.3 (1.4)	8.2 (1)	< 0.001^*^	5.8 (1.4)	8.2 (1.1)	< 0.001^*^
MIDAS category	No disability	0	0	0.797	2 (12.5%)	0	0.106	4 (25%)	0	0.021^*^
	Mild	2 (12.5%)	4 (21.1%)		5 (31.3%)	4 (21.1%)		4 (25%)	4 (21.1%)	
	Moderate	2 (12.5%)	2 (10.5%)		4 (25%)	2 (10.5%)		5 (31.3%)	3 (15/8%)	
	Severe	12 (75%)	13 (68.4%)		5 (31.3%)	13 (68.4%)		3 (18.8%)	12 (63.2%)	
HIT		63.7 (5.5)	65.6 (6.6)	0.357	56.9 (6.9)	62.4 (9.2)	0.099	53.4 (6)	63.7 (7.8)	< 0.001^*^
HIT category	Not severe	0	1 (5.3%)	0.352	4 (25%)	3 (15.8%)	0.497	8 (50%)	1 (5.3%)	0.003^*^
	Severe	16 (100%)	18 (94.7%)		12 (75%)	16 (84.2%)		8 (50%)	18 (94.7%)	
HADS-anxiety		10.5 (4.5)	10.5 (3.2)	0.642	7.9 (4.3)	10.6 (3.7)	0.002^*^	6.8 (3.6)	11.1 (3)	< 0.001^*^
HADS-anxiety category	Normal	4 (25%)	4 (21.1%)	0.959	10 (62.5%)	6 (31.6%)	0.092	10 (62.5%)	3 (15.8%)	0.013^*^
	Borderline	5 (31.3%)	6 (31.6%)		0	3 (15.8%)		3 (18.8%)	5 (26.3%)	
	Abnormal	7 (43.8%)	9 (47.4%)		6 (37.5%)	10 (52.6%)		3 (18.8%)	11 (57.9%)	
HADS-depression		9.8 (3.5)	10.3 (3.7)	0.684	7.8 (2.8)	10.6 (2.9)	< 0.001^*^	7.5 (2.1)	11.2 (3.2)	< 0.001^*^
HADS-depression category	Normal	5 (31.3%)	6 (31.6%)	0.511	10 (62.5%)	3 (15.8%)	0.017^*^	9 (56.3%)	3 (15.8%)	0.011^*^
	Borderline	5 (31.3%)	3 (15.8%)		3 (18.8%)	8 (42.1%)		5 (31.3%)	5 (26.3%)	
	Abnormal	6 (37.5%)	10 (52.6%)		3 (18.8%)	8 (42.1%)		2 (12.5%)	11 (57.9%)	
Emergency department visits for headache per month (number of cases)		2 (12.5%)	3 (15.8%)	0.782	1 (6.3%)	1 (5.3%)	0.900	0	1 (5.3%)	0.352

*VAS* Visual Analog Scale, *MIDAS* Migraine Disability Assessment Scale, *HIT* Headache Impact Test, *HADS* Hospital Anxiety and Depression Scale, *Days* Number of days the patients have experienced headache within the past 30 days, *Pain relievers* Number of pain-relievers taken during the past 30 days

^1^Between group comparison. *P*-value calculated using Chi-square test (for categorical variables) or independent t-test (for interval variables) as appropriate

^2^Between group comparison. *P*-value calculated using Chi-square test (for categorical variables) or one-way analysis of covariance (for interval variables) after adjusting for baseline scores

^*^Indicating statistically significant *p*—values

### In-group comparisons

We used repeated measure ANOVA test to evaluate changes in outcome measures and compare the values between different study time-points. Based on these findings, we observed statistically significant differences in all categories (VAS, MIDAS, HIT, HADS, headache days, and pain-relievers) between study time-points in the intervention group (*p* < 0.05). However, we found no similar statistically significant differences in the control group (*p* > 0.05).

Post-hoc analysis of the results in the intervention group using Bonferroni test is presented in Table 4. According to the table, there were statistically significant differences between the baseline scores/values and those scores from 3-month and 4-month follow-ups in all outcome measures (*p* < 0.05). Moreover, no statistically significant differences were found between 3-month and 4-month follow-ups in MIDAS-B, HADS-depression, and number of pain-relievers (*p* > 0.05), indicating a persistent effect of the intervention. Other outcome measures showed continued declined scores in the 4-month follow-up with statistically significant differences compared to the 3-month follow-up scores (*p* < 0.05), indicating persistent improvement.

**Table 4:** Pairwise comparison of outcome measures between different study time-points in the intervention group

**Category**	**Time-point**	**Reference time-point**	**Mean Difference**	**Standard Error**	***P*-value**	**95% Confidence Interval for Difference**
Visual analog scale (headache severity)	1	2	1.875	0.340	< 0.001	(1.150, 2.600)
		3	2.500	0.342	< 0.001	(1.772, 3.228)
	2	3	0.625	0.352	0.096	(-0.125, 1.375)
Migraine disability assessment scale (MIDAS)	1	2	21.875	4.513	< 0.001	(12.256, 31.494)
		3	23.813	4.841	< 0.001	(13.494, 34.131)
	2	3	1.938	0.692	0.013	(0.462, 3.413)
MIDAS-A	1	2	16.938	3.269	< 0.001	(9.970, 23.905)
		3	19.563	3.217	< 0.001	(12.705, 26.420)
	2	3	2.625	1.197	0.044	(0.074, 5.176)
MIDAS-B	1	2	1.594	0.369	0.001	(0.808, 2.379)
		3	2.063	0.413	< 0.001	(1.182, 2.943)
	2	3	0.469	0.314	0.157	(-0.202, 1.139)
Headache impact test (HIT)	1	2	6.750	1.493	< 0.001	(3.568, 9.932)
		3	10.313	1.128	< 0.001	(7.908, 12.717)
	2	3	3.563	1.151	0.007	(1.109, 6.016)
Hospital anxiety and depression scale-Anxiety	1	2	2.625	0.774	0.004	(0.975, 4.275)
		3	3.688	0.762	< 0.001	(2.063, 5.312)
	2	3	1.063	0.403	0.019	(0.204, 1.921)
Hospital anxiety and depression scale-Depression	1	2	2.063	0.433	< 0.001	(1.140, 2.985)
		3	2.313	0.561	0.001	(1.118, 3.507)
	2	3	0.250	0.487	0.615	(-0.789, 1.289)
Days with headache within the past 30 days	1	2	5.625	1.179	< 0.001	(3.111, 8.139)
		3	8.125	1.541	< 0.001	(4.841, 11.409)
	2	3	2.500	1.114	0.040	(0.125, 4.875)
Number of pain-relievers taken during the past 30 days	1	2	7.188	1.085	< 0.001	(4.875, 9.500)
		3	8.688	1.413	< 0.001	(5.675, 11.700)
	2	3	1.500	0.764	0.068	(-0.128, 3.128)

Results based on estimated marginal means

Comparing the categorized MIDAS, HIT, HADS-anxiety, and HADS-depression in the intervention group between three time-points showed statistically significant differences between these frequencies (*p* < 0.001, *p* = 0.005, *p* = 0.003, and *p* = 0.005, respectively). Similar analysis in the control group for MIDAS, HIT, HADS-anxiety, and HADS-depression showed no statistically significant differences (*p* = 0.368, *p* = 0.264, *p* = 0.229, and *p* = 0.326, respectively).

## Discussion

In the present study, we investigated the efficacy of TCBT in PwM. TCBT showed favorable effects on decreasing days of headache, headache severity, migraine related disability, migraine effects on daily life, number of pain-relievers used for headache, depression, and anxiety. These effects were also present after one month of therapy discontinuation indicating persistency of the effects.

A systematic review of 24 studies showed the efficacy of psychological interventions in migraine [10]. Behavioral therapies with different approaches are among the most suggested psychological interventions for PwM [9]. Despite the observed efficacy in research setting, a gap in practice exists in delivering mental health services [25, 26]. Multiple factors have been suggested to contribute to this gap, including cost, patient willingness to pursue psychological interventions, adherence to therapy, and availability of trained personnel. With regards to patient willingness, a recent study indicated tendency in pursuing behavioral therapy among PwM especially in those with moderate to severe level; however, patients were not willing to pay for the therapy sessions when they did not have insurance coverage for such treatments [12].

While in-person behavioral therapy sessions cost considerable amounts for both the patient and health care system, implementing group-based sessions are less expensive and more practical. The transdiagnostic nature of TCBT means this technique can be used in a group-based setting with individuals suffering from different types of disorders and comorbidities [14, 15, 27]. This would further facilitate the process for therapists and centers delivering this service to enroll enough number of cases for a group. Given these characteristics, TCBT is more favorable for both the patient and health care delivery system [13, 27]. We designed the therapy modules within 10 sessions of two hours, as brief therapy modules have been suggested to increase feasibility of delivering care and patient adherence with favorable outcomes [28].

In the study by Sharma et al. in 2017, 63 adolescents suffering concurrent anxiety and primary headache (episodic tension-type, migraine, or cluster headache) were divided into two groups of intervention and control. The intervention group underwent 12 weeks of group based TCBT while the control group only continued their previous pharmacotherapy. Improvements in the Headache Impact Test and Global Assessment Scale for Children were observed at the end of the study in both groups; however, results from the TCBT group were better than the control group [15]. Also, improvement in the State Trait Anxiety Inventory was only observed in the TCBT group and not the control group. Of note, the study was limited by different baseline features of two groups, favoring the intervention group [15].

In the study by Klan et al. the efficacy of an integrative CBT program was assessed among 9 adults suffering migraine [29]. The program consisted of 7 sessions, each lasting for 90 min. Patients reported a high satisfaction with therapy sessions, and the treatment integrity assessment and the qualitative interview by the patients were favorable as well. However, no statistically significant improvements in pre-treatment and post-treatment comparisons of the following tests were observed: The Headache Disability Inventory, The Pain Disability Index, Headache Impact Test, Depression Anxiety Stress Scales, Headache Management Self-Efficacy Scale-short form, Headache Triggers Sensitivity and Avoidance Questionnaire, and Chronic Pain Acceptance Questionnaire [29]. The small number of participants has possibly been the main reason for lack of improvement in observed scores.

In another study, Sajadinejad et al. investigated the efficacy of 9 weeks of group-based CBT (one session each week) on 20 female patients suffering tension type headache or migraine. Researchers used headache disability inventory and Beck depression inventory before intervention, after 5 weeks of therapy and at the last therapy session. They reported improvements in headache disability and depression in the assessments performed after 5 and 9 weeks of therapy. Worth mentioning that the authors failed to report headache characteristics and its associated changes among their patients [30]. Neither the study by Klan et al. nor the study by Sajadinejad et al. had a control group for comparison.

In the current study, we found improved quantitative scores in all used questionnaires at the time of therapy termination (three-month follow-up), except for HIT score Also, the categorical MIDAS, HIT, and HADS-anxiety were not different between two groups, with only HADS-depression showing improvement. One month after therapy termination (four-month follow-up), all scores and categories of the mentioned outcome measures were improved in the intervention group compared to the control group. These findings indicate favorable outcome of TCBT among PwM in improving headache and associated disabilities, as well as concomitant anxiety and depression.

Regarding the persistency of behavioral therapy effects, it has been reported that the beneficial effects of CBT in patients suffering headaches and migraine would decline within several weeks of therapy termination [11]. Here we did not find a decline on the therapeutic effects of TCBT based on the assessments performed one month after therapy termination. In-group comparison of outcome measures in the intervention group showed no change or improvement of outcomes from three-month to four-month follow-up, implying the short-term persistency of our intervention. Our study design, however, does not allow us to evaluate the persistency of therapy effects on longer follow-up periods. Moreover, our therapy sessions were more focused on stress management, problem solving, distraction techniques, and negative thoughts, possibly leading to a stronger effect on anxiety and migraine-induced disability. Looking at 4-month vs 3-month scores shows that patients improved in how to live with their migraine more efficiently and manage their anxiety, and this effect persistent 4 weeks after therapy termination.

To have a better understanding of our findings, we need to see the demographics of our recruited cases: young females with a graduate degree in more than half of cases, with severe migraine, severe associated disability, and abnormal levels of anxiety and depression in most cases. We believe these patients were more likely to participate in the study given the level of distress they are bearing, compared to those with mild to moderate severity or disability and more controllable migraine. While our cases are probably not representative of all PwM, they show how effective TCBT could be in severe migraine cases. Moreover, anxiety and depression are known as precipitating factors for migraine that could lead to disease progression [31, 32]. Therefore, interventions affecting these factors could ultimately lead to a better patient-reported outcome. We speculate that changes in patients’ perspectives toward their daily lives and the accompanying anxieties have helped them to reconsider their habitude in dealing with problems. We think that TCBT have helped patients to strengthen their ability in dealing with their headache through new skills on controlling stress and coping with pain and distressing situations. Hence, their feelings of disability were significantly reduced, and they were able to implement the new learned skills in their lives, leading to the persistency of effects.

There are limitations to the present study. First, patients in the control group only underwent one session of therapy, while those in the intervention group had ten sessions. The more time spent with the therapist eventually may result in placebo effect regardless of the intervention. Moreover, given the nature of our intervention, we were not able to mask patients or our therapist of the randomization status. Therefore, the current study design would not eliminate the placebo effect completely considering the longer duration of therapy in the intervention group and their engagement with the therapy by working on their assignments. Second, most of our participants were young females. Considering that migraine is more prevalent among women and that women are more willing to pursue psychological interventions, this was inevitable in our recruitment process. Third, we were not able to follow up patients for more than one-month after therapy termination, given the limited funds and personnel. Therefore, we are not able to evaluate the long-term persistency of TCBT among PwM. Fourth, we did not ask our patients to complete satisfaction surveys after each session of therapy. But they were asked to give verbal feedback at the end of each session, and they reported high levels of satisfaction. Despite these limitations, to the best of our knowledge this is the first study evaluating the efficacy of TCBT on adult PwM, compared to a control group receiving limited sham therapy session. Moreover, we provided a short-term follow-up evaluation which has not been done in many of the previous studies. Future studies with larger sample sizes, equal number of sham therapy sessions to the intervention sessions, a more diverse patient population, and longer follow-ups are needed to confirm our findings.

## Conclusion

Based on our findings, TCBT could be an effective intervention for PwM in lowering migraine severity, decreasing the associated disability, and improving anxiety and depression. Furthermore, TCBT might have short-term benefits in this group of patients. Of note, due to limitations in the study design, these findings need to be confirmed in future studies with better control for placebo effect. TCBT is a is a feasible, practical, and cost-effective technique, and could be used along with the routine medication therapy in PwM.

### Clinical implications

Transdiagnostic cognitive behavioral therapy (TCBT) improves migraine severity.

TCBT decreases migraine-associated disability and improves comorbid anxiety and depression in people with migraine.

TCBT is a feasible, practical, and cost-effective technique to be used in people with migraine.

### Public health relevance

Despite the reported efficacy of psychological interventions in various medical conditions, these interventions are not widely used in real practice. Effective, brief interventions could facilitate the utilization of these techniques.

## Data Availability

The data that support the findings of this study are available from Islamic Azad University of Khomeinishahr but restrictions apply to the availability of these data, which were used under license for the current study, and so are not publicly available. Data are however available from the authors upon reasonable request and with permission of Islamic Azad University of Khomeinishahr.
